# Illumina short-read and MinION long-read WGS to characterize the molecular epidemiology of an NDM-1 *Serratia marcescens* outbreak in Romania

**DOI:** 10.1093/jac/dkx456

**Published:** 2017-12-08

**Authors:** H T T Phan, N Stoesser, I E Maciuca, F Toma, E Szekely, M Flonta, A T M Hubbard, L Pankhurst, T Do, T E A Peto, A S Walker, D W Crook, D Timofte

**Affiliations:** 1 Modernising Medical Microbiology Consortium, Nuffield Department of Clinical Medicine, University of Oxford, Oxford, UK; 2 National Institute for Health Research (NIHR) Health Protection Research Unit (NIHR HPRU) in Healthcare Associated Infections and Antimicrobial Resistance, University of Oxford, Oxford, UK; 3 Institute of Veterinary Science, University of Liverpool, Leahurst Campus, UK; 4 Microbiology Department, University of Medicine and Pharmacy, Targu Mures, Romania; 5 Clinical Hospital of Infectious Diseases, Cluj-Napoca, Romania; 6 Institute of Infection and Global Health, University of Liverpool, Liverpool, UK

## Abstract

**Background and Objectives:**

*Serratia marcescens* is an emerging nosocomial pathogen, and the carbapenemase *bla*_NDM_ has been reported in several surveys in Romania. We aimed to investigate the molecular epidemiology of *S. marcescens* in two Romanian hospitals over 2010–15, including a neonatal NDM-1 *S. marcescens* outbreak.

**Methods:**

Isolates were sequenced using Illumina technology together with carbapenem-non-susceptible NDM-1-positive and NDM-1-negative *Klebsiella pneumoniae* and *Enterobacter cloacae* to provide genomic context. A subset was sequenced with MinION to fully resolve NDM-1 plasmid structures. Resistance genes, plasmid replicons and ISs were identified *in silico* for all isolates; an annotated phylogeny was reconstructed for *S. marcescens*. Fully resolved study NDM-1 plasmid sequences were compared with the most closely related publicly available NDM-1 plasmid reference.

**Results:**

44/45 isolates were successfully sequenced (*S. marcescens*, *n *=* *33; *K. pneumoniae*, *n *=* *7; *E. cloacae*, *n *=* *4); 10 with MinION. The *S. marcescens* phylogeny demonstrated several discrete clusters of NDM-1-positive and -negative isolates. All NDM-1-positive isolates across species harboured a pKOX_NDM1-like plasmid; more detailed comparisons of the plasmid structures demonstrated a number of differences, but highlighted the largely conserved plasmid backbones across species and hospital sites.

**Conclusions:**

The molecular epidemiology is most consistent with the importation of a pKOX_NDM1-like plasmid into Romania and its dissemination amongst *K. pneumoniae*/*E. cloacae* and subsequently *S. marcescens* across hospitals. The data suggested multiple acquisitions of this plasmid by *S. marcescens* in the two hospitals studied; transmission events within centres, including a large outbreak on the Targu Mures neonatal unit; and sharing of the pKOX_NDM1-like plasmid between species within outbreaks.

## Introduction

Carbapenemase-producing Enterobacteriaceae (CPE) have emerged post-1990 and spread worldwide, representing a serious clinical threat.^1^ Although carbapenem resistance can arise through various mechanisms, acquisition of genes (*bla*_KPC_, *bla*_NDM-1_, *bla*_OXA-48_) encoding *Klebsiella pneumoniae* carbapenemase (KPC), New Delhi metallo-β-lactamase (NDM) and OXA-48 carbapenemase, respectively, are currently of most concern. These genes are readily transmitted intra- and inter-species via mobile genetic elements that have facilitated their worldwide dissemination.^2^

Compared with *Escherichia coli* and *K. pneumoniae*, two of the most frequently identified CPE species, *Serratia marcescens* has been less commonly associated with any of the major transmissible carbapenemases, although acquisition of *bla*_KPC_ has been described recently in China, Greece and the United States.^3–5^*S. marcescens* has also traditionally been regarded as an opportunistic pathogen causing disease in specifically vulnerable populations, such as neonates.^6^^,^^7^ However, *Serratia* spp. have become increasingly adapted to hospital environments and have emerged as important agents of hospital-acquired infections affecting all age groups.^8–10^ The inherent resistance of *Serratia* spp. to multiple antimicrobial classes may have facilitated this adaptation, and leaves few therapeutic options open for treating carbapenem-resistant *S. marcescens* infections.^11–13^

*bla*_NDM-1_ has been increasingly reported in patients with healthcare exposure on the Indian subcontinent or in the Balkan region.^1^^,^^14^^,^^15^ The occurrence of *bla*_NDM-1_ in *S. marcescens* appears rare, with only a handful of isolates identified in Germany,^16^ the UK^17^ and Egypt.^18^ However, a recent faecal screening/surveillance project for carbapenemase-producing Gram-negative bacteria in three Romanian hospitals identified 17 NDM-1 *S. marcescens* in clinical, environmental and screening isolates,^19^ all of which were from the same neonatal unit at a single hospital, consistent with an outbreak. PCR-based plasmid analyses identified *bla*_NDM-1_ in association with conjugative IncFII plasmids in both *S. marcescens* and *K. pneumoniae*, suggesting inter-species transfer of *bla*_NDM-1_, possibly from *K. pneumoniae* into a well-established hospital-adapted *S. marcescens* clone.

In this study, we used Illumina (short-read) and MinION (long-read) WGS of NDM-1- and non-NDM-1 *S. marcescens* and other Enterobacteriaceae isolates obtained from: (i) the faecal screening/surveillance study; (ii) a previous outbreak and sporadic *S. marcescens* infections in this neonatal unit; and (iii) a second hospital in an adjacent county, to investigate the evidence for nosocomial and inter-hospital dissemination of NDM-1 *S. marcescens* and the transmission of NDM-1 plasmids amongst Enterobacteriaceae in this context in Romania.

## Materials and methods

### Hospital settings

Bacterial isolates were collected from two teaching hospitals located in central/north Romania: one in Targu Mures (1099 beds, ∼42 000 patients/year), and one in Cluj-Napoca (252 beds, ∼20 000 patients/year). In Targu Mures, the neonatology unit had 65 beds [10 were for neonatal intensive care (NICU), 15 for the care of premature neonates].

### Overview of S. marcescens outbreaks in Targu Mures hospital

During April–June 2010, 11 neonates were found to be colonized (faecal/pharyngeal) with carbapenem-susceptible *S. marcescens*, 4 of whom (36%) developed *S. marcescens* bacteraemia. Environmental screening did not identify any source; PFGE (data not shown) was consistent with transmission of a clonal strain with the main vector presumed to be colonized neonates. The main contributing factors to carbapenem-susceptible *S. marcescens* dissemination were thought to be overcrowding in the NICU and selective pressure exerted by ampicillin/sulbactam and gentamicin prophylaxis given to high-risk neonates (premature and/or complicated delivery). All patients survived and the outbreak was declared over in July 2010.

Sporadic infections with carbapenem-susceptible *S. marcescens* recurred in 2012, whilst both colonization events and infections with carbapenem non-susceptible-*K. pneumoniae* and carbapenem non-susceptible *E. cloacae* occurred during 2012–13 in the NICU and paediatric cardiology ward. Following this, in August 2014, several cases of carbapenem non-susceptible *S. marcescens* pharyngeal colonization occurred in the neonatology unit and an outbreak was declared in October 2014; enhanced infection control measures were implemented, including faecal screening of all admissions/transfers to the unit. At this point, the genetic mechanism mediating carbapenem resistance was shown by PCR to be *bla*_NDM-1_. Six cases of NDM-1 *S. marcescens* bacteraemia occurred October–December 2014; ongoing NDM-1 *S. marcescens* faecal/pharyngeal colonization cases were identified up until May 2015.

In both outbreaks in the Targu Mures NICU (2010, 2014–15), infection control measures were reinforced, consisting initially of isolating colonized/infected patients and their contacts, terminal cleaning with quaternary ammonium compounds, and special attention to NICU disinfection. At the same time, hospital staff were re-educated with respect to contact precautions and hand hygiene procedures to try and reduce the risk of any staff–patient transmission.

### Isolates

#### Targu Mures hospital isolates

Isolates from Targu Mures included those obtained from faecal screens performed during active carbapenemase surveillance in the 2014–15 *S. marcescens* outbreak, as well as any clinical carbapenem-non-susceptible *S. marcescens* isolated from the neonatal unit. Other carbapenem-non-susceptible *S. marcescens*, carbapenem-non-susceptible *K. pneumoniae* and carbapenem-non-susceptible *E. cloacae* obtained from clinical specimens on other hospital units were also included for comparison.

Carbapenem-non-susceptible *S. marcescens* cultured from environmental screening samples (October 2014–April 2015); previous NDM-1 *K. pneumoniae* and NDM-1 *E. cloacae* isolated from clinical specimens; and carbapenem-susceptible *S. marcescens* obtained from a previous outbreak in the same neonatal unit (2010) and from sporadic clinical cases (2012) were also included in the study. Ethics approval for surveillance was obtained from the Hospital Research Ethics Committee (#138; 23/12/2013).

#### Cluj-Napoca hospital isolates

Cluj-Napoca is a city neighbouring Targu Mures (∼80 km away from Targu Mures) and patients travel between these cities for medical intervention not provided locally. A subset of stored *S. marcescens* isolates with a carbapenemase-producing phenotype (ertapenem- and meropenem-resistant and CarbaNP test-positive) from urine samples were randomly selected to identify evidence for inter-hospital transmission.

### Microbiological methods

For faecal screening, a modified method from the US CDC involving enrichment and subculture on selective media [trypticase soy broth (TSB)/MacConkey + carbapenem] was used.^20^ In brief, a rectal swab or a small amount of faecal sample was placed in 5 mL of TSB containing a meropenem (10 μg) disc and incubated at 35 ± 2^°^C overnight. 100 μL of inoculated TSB was subsequently streaked onto MacConkey agar and carbapenem discs (ertapenem 10 μg and meropenem 10 μg) were placed on different sectors of the inoculum (all discs and media from Oxoid, Basingstoke, UK); screening cut-off values for CPE were applied according to the EUCAST methodology (v5.0, available at: http://www.eucast.org/fileadmin/src/media/PDFs/EUCAST_files/Breakpoint_tables/v_5.0_Breakpoint_Table_01.pdf). Presence of carbapenemases was confirmed phenotypically using the CARBA NP test (BioMérieux, Paris, France) at Cluj-Napoca or by PCR (for *bla*_NDM_, *bla*_KPC_, *bla*_IMP_ and *bla*_VIM_) at Targu Mures.^21^^,^^22^ Clinical specimens were processed according to local protocols. Species identification was performed using the VITEK 2 (BioMérieux); susceptibility testing was performed by microbroth dilution (TREK Diagnostic Systems, West Sussex, UK) for isolates from the faecal surveillance study and by disc diffusion for the others (EUCAST guidelines).

### DNA extractions and Illumina/MinION sequencing

DNA extraction of sub-cultured isolates for Illumina sequencing was performed using the Quickgene commercial kit (Fujifilm, Tokyo, Japan), with an additional mechanical lysis step following chemical lysis (FastPrep, MP Biomedicals, Santa Ana, USA). Isolates were sequenced using the Illumina HiSeq 2500 (150 bp paired-end reads; sequencing coverage ∼100×).

Ten isolates were selected for MinION sequencing based on Illumina data; isolates were cultured from frozen stocks (−80 °C) on Columbia Blood agar in the presence of three ertapenem discs (10 μg; Oxoid, Thermo Fisher Scientific, USA) overnight at 37 °C. DNA was then extracted using the Qiagen Genomic tip 100/G kit as per the manufacturer’s instructions (Qiagen, Venlo, the Netherlands), quantified using the Qubit 2.0 Fluorometer (Life Technologies, USA), and fragment lengths assessed using the TapeStation 2200 (Agilent, UK).

Library preparation was performed using the 2D native barcoding protocol, targeting 8 kb fragments, and with an input of 3–4 μg DNA. All steps were performed following the manufacturer’s protocol with native barcoding to multiplex two samples per sequencing run (EXP-NBD002 and SQK-LSK208). Libraries were sequenced using the best available flow cells at the time (R9.4). Libraries were topped up after 12 h, and sequencing performed for 48 h in total. Data were base-called in real time via Metrichor, using the best available workflow at the time (v1.125; November–December 2016 and January 2017; ONT, UK). Insufficient sequence data yields were generated for two samples (multiplexed in a single run; 7209 and 18ES), requiring repeat sequencing of the same DNA extract by the same methods. The sequence data from both runs were merged to generate a final set of data for further processing.

#### WGS analysis

For Illumina data, read quality- and adapter-related trimming and filtering were performed using BBDuk (BBTools; available at https://jgi.doe.gov/data-and-tools/bbtools/), and species identification was confirmed *in silico* on the basis of the top species match identified using Kraken.^23^ The phylogeny of *S. marcescens* isolates was reconstructed using IQTree^24^ from a consensus fasta of variant sites generated from mapping to the *S. marcescens* reference WW4 (NC_020211.1). Read mapping was performed using Stampy,^25^ with mapping and filtering of variant calls based on a set of thresholds related to base and mapping quality and read coverage, as previously described.^26^ For phylogenetic reconstruction, a General Time Reversible model^27^ was used with a gamma category allowing variable mutation rates across sites, and a maximum parsimony starting tree. The maximum-likelihood phylogeny output by IQTree was then corrected for recombination using ClonalFrameML^28^ (default parameters), and visualized in iTOL.^29^

*De novo* assemblies of short-read sequenced isolates were generated with SPAdes (v3.6; default settings).^30^ We used an arbitrary assembly size of >6.5 Mb as a threshold to identify any mixed sequences (including mixtures of the same species not identified by Kraken) and remove them from the analysis. *In silico* multi-locus sequence-typing (MLST) for *K. pneumoniae* and *E. cloacae* isolates was performed using BLASTn of the sequence assemblies against the MLST allele databases of these two species downloaded from https://pubmlst.org/general.shtml. Plasmid replicon typing was performed using BLASTn against the PlasmidFinder database,^31^ and IS typing using the ISFinder database.^32^ Resistance gene characterization was performed using the in-house script ResistType, a BLASTn/local reassembly-based approach for querying the presence/absence of known chromosomal and acquired resistance mechanisms using a database as described in Stoesser *et al.*^33^ (scripts and database available at: https://github.com/hangphan/resistType_docker). This database includes >2000 variants from >70 resistance gene families, covering all the major resistance gene mechanisms and many minor ones.

NDM-1 plasmid typing was performed using a reference database of NDM-1 plasmids generated in this study and the subset harbouring NDM-1 from a curated database of plasmids downloaded from NCBI.^34^ BLASTn was then used to query the sequence assemblies for the presence of similar *bla*_NDM_-carrying plasmid structures. An isolate was characterized as having candidate NDM-1-plasmid ‘X’ if >90% of plasmid ‘X’ could be aligned with the query assembly’s contigs, where for each contig, >90% of the contig length matched plasmid ‘X’ with >95% sequence identity. Thresholds were set so that an isolate could have multiple matches to the NDM-1 plasmid database.

Poretools^35^ was used to extract 2D reads from base-called MinION sequencing data and Canu (v1.4)^36^ to generate *de novo* assemblies, which were error-corrected using short-read Illumina data and the pilon assembly polisher (v1.18).^37^ A plasmid was defined as circularized/complete if it had >1 kb overlapping ends with >99% sequence identity. Alignments of fully reconstructed plasmid sequences were visualized and annotated in Geneious (version: R9).^38^

#### Data accession

All short-read sequencing data (fastq files) and long-read sequencing data (assemblies) from the study are available in GenBank (BioProject accession: PRJNA396838).

## Results

### Bacterial isolates

Four hundred and seventeen faecal/pharyngeal (*n *=* *135), staff hand swabs (*n *=* *90) and environmental samples (*n *=* *192) were collected from the neonatology unit during the Targu Mures surveillance study, which overlapped with the major *S. marcescens* outbreak period (January–May 2015). From these, 24 carbapenem-non-susceptible Enterobacteriaceae were cultured (*S. marcescens*, *n *=* *19, *K. pneumoniae*, *n *=* *5; 19/19 *S. marcescens* were found by PCR to carry *bla*_NDM-1_; 5/5 *K. pneumoniae* were Carba NP test-positive but not tested by PCR). Positive cultures were obtained from faecal screens (*n *=* *14) and pharyngeal secretions (*n *=* *7) from 14 neonates/children, and three environmental samples (one from a bed sheet, two from feeding tubes). NDM-1 *S. marcescens* neonatal bacteraemia isolates from the 2014–15 outbreak (*n *=* *5), carbapenem-susceptible *S. marcescens* from an earlier outbreak (2010, *n *=* *3) and sporadic carbapenem-susceptible *S. marcescens* cases (2012, *n *=* *3), as well as previous sporadic NDM-1 *K. pneumoniae* (*n *=* *3, 2013) and NDM-1 *E. cloacae* (*n *=* *3, 2013) were included. From Cluj-Napoca, five carbapenem non-susceptible *S. marcescens* were obtained for comparison. One sample was lost in processing, resulting in 45 sent for WGS (Table S1, available as Supplementary data at *JAC* Online).

### WGS data analysis

**Figure 1.**
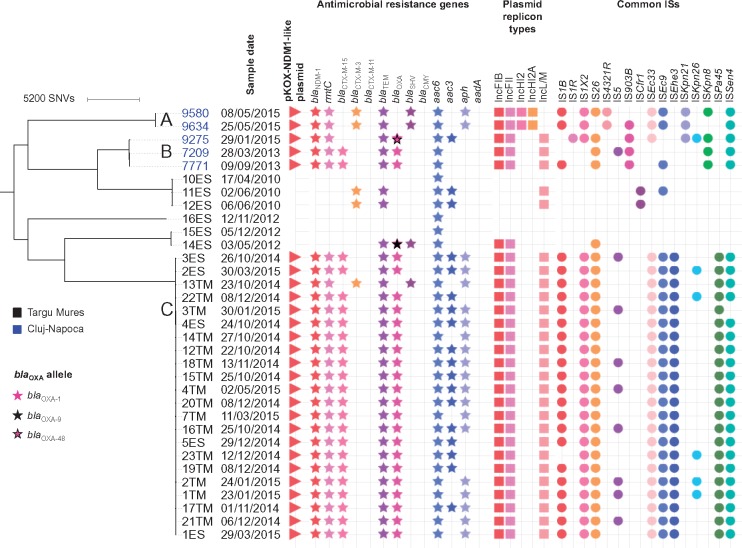
**Phylogeny of the sequenced *Serratia marcescens* strains analysed in this study, annotated by location, sampling date and genomic features.** Genetically related clusters of NDM-1-positive isolates are denoted by the letters A–C.

Forty-four of the 45 sequences passed assembly quality control, including *K. pneumoniae* (*n *=* *7; 2 new ST, 3 ST-15, 1 ST-45, 1 ST-307), *E. cloacae* (*n *=* *4; 2 ST-90, 1 ST-254, 1 ST-104) and *S. marcescens* isolates (*n *=* *33; 27 NDM-1-positive; Figure S1). The earliest confirmed NDM-1-positive strains of each species were *S. marcescens* 7209 (28/03/2013) in Cluj-Napoca and in Targu Mures *E. cloacae* 20ES (27/07/2013), *K. pneumoniae* 19ES (07/08/2013), and *S. marcescens* 12TM (22/10/2014). Phylogenetic analysis of *S. marcescens* isolates was consistent with a clonal outbreak of NDM-1 *S. marcescens* across clinical, environmental and faecal screening isolates in the Targu Mures neonatal/paediatric units [<25 single nucleotide variations (SNVs) distance across all isolates, 2014–15]. This clonal strain was however chromosomally distantly related (>3300 SNVs) to the 2010–12 NDM-1-negative *S. marcescens* isolates from Targu Mures and the 2013–15 NDM-1-positive *S. marcescens* isolates from Cluj-Napoca (Figure 1). Other antimicrobial resistance genes were common (Figure 1); two isolates, *K. pneumoniae* 17ES from Targu Mures and *S. marcescens* 9275 from Cluj-Napoca, harboured both *bla*_NDM-1_ and *bla*_OXA-48_.

**Figure 2.**
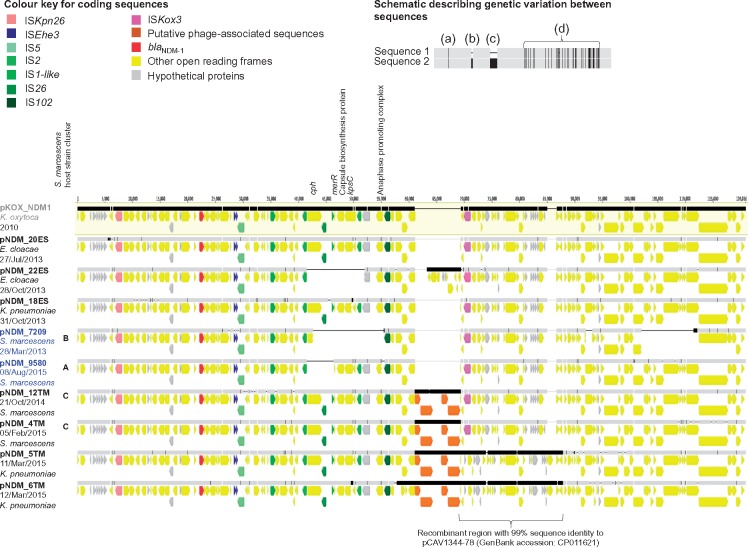
**Alignment of study NDM-1 plasmid sequences and the reference NDM-1 plasmid pKOX_NDM1.** Aligned bars adjacent to plasmid names represent plasmid sequences: light grey denotes regions with 100% sequence identity; black represents nucleotide diversity between sequences; and thin lines represent indels. Coding sequences are represented by fat arrows below individual sequence bars and are colour coded as per the colour key. The inset schematic describing genetic variation between sequences depicts examples of evolutionary events identified: (a) single nucleotide level change, (b) small indels (≤100 bp), (c) large indels (>100 bp), (d) recombination events. Sequence labels are coloured by location (grey, reference; black, Targu Mures hospital; blue, Cluj-Napoca hospital). The letters A–C correspond to isolate clusters in Figure 1, and represent the relatedness of the host strain in which the NDM-1 plasmids were found.

Long-read sequencing generated 12–98× coverage for the 10 isolates sequenced (Figure S2), confirmed the absence of *bla*_NDM_ in one *S. marcescens* isolate (14ES, 2012), and enabled closure of the NDM-1 plasmid sequences (87.5–120 kb) in the other nine isolates [*S. marcescens* (*n *=* *4), *K. pneumoniae* (*n *=* *3) and *E. cloacae* (*n *=* *2); Table S2]. All nine NDM-1 plasmids shared high sequence similarity with pKOX_NDM1 (accession number NC_021501.1), an IncFII_Y_ plasmid first identified in Taiwan in 2010^39^ (Figure 2). Several genetic changes with respect to pKOX_NDM1 were observed in the study plasmids, including: scattered nucleotide mutations; clusters of nucleotide mutations consistent with small recombination events; and small and large indels. Examples of indels included: (i) deletions of all or part of a cluster of genes including *cph* (encoding for a bacterial phytochrome), *merR* (regulator of the *mer* operon encoding for mercury resistance) and the capsular polysaccharide biosynthesis operon (including *kpsC*), the latter two of which have been associated with antimicrobial resistance, environmental persistence and/or virulence^40^^,^^41^ (pNDM_22ES, pNDM_7209, pNDM_9580); (ii) deletions of several open reading frames involved in conjugal transfer (pNDM_7209); and (iii) acquisition of a set of putative phage-associated elements in neonatal outbreak isolates from 2014 to 2015 (pNDM_12TM, pNDM_4TM, pNDM_5TM, pNDM_6TM) (Figure 2). A large recombination event was also observed in pNDM_5TM and pNDM_6TM, encompassing the region downstream of the putative phage to *traM* (Figure 2), and genetically highly similar (99%) to ∼18 kb of another reference plasmid, pCAV1344-78 (GenBank accession: CP011621, Charlottesville, USA). This region contained two *klcA* paralogues; *klcA* encodes for an anti-restriction protein, which has recently been shown to promote horizontal gene transfer amongst KPC plasmids.^42^

Plasmid typing of the other isolates (*S. marcescens*, *K. pneumoniae* and *E. cloacae*) using short-read Illumina data was consistent with pKOX_NDM1-like plasmids being present in all NDM-1-positive strains, although the context and structure of these plasmids could not be resolved from the Illumina assemblies. There were no significant matches to any non-pKOX_NDM1 NDM-1-harbouring plasmids (Figure S1; all NDM-1-harbouring isolates have a top match to pKOX_NDM1).

## Discussion

Here, we show that the molecular epidemiology of carbapenemase-non-susceptible *S. marcescens* in two regional Romanian hospitals is consistent with the emergence of multiple NDM-1 *S. marcescens* strain clusters in conjunction with pKOX_NDM1-like plasmids, causing clinical outbreaks over 2013–15. NDM-1 *S. marcescens* was isolated from both asymptomatic neonates and a small number of environmental samples, and caused high rates of invasive disease (35%) in this susceptible population. Highly genetically similar pKOX_NDM1-like plasmids were observed in contemporaneously circulating *E. cloacae* and *K. pneumoniae*, suggestive of a multi-species, multi-hospital pKOX_NDM1-like plasmid transmission network, and acquisition of this plasmid by genetically distinct strains of *S. marcescens* in Targu Mures and Cluj-Napoca hospitals, causing the observed outbreak in Targu Mures. Staff and environmental sampling failed to identify a major non-patient reservoir, as elsewhere.^10^ Although most NDM-1 *S. marcescens* isolates were obtained in the context of the neonatal outbreak, several NDM-1 *S. marcescens* isolates were from clinical specimens obtained from adults, suggesting that these isolates are also important to consider in non-neonatal patient groups.

There are limited data on carbapenemase-producing Enterobacteriaceae in Romania, but the earliest published reports of NDM-1-producing Enterobacteriaceae in this country are from Targu Mures hospital, one of our study hospitals. Surveillance between January 2010 and September 2012 identified 9/2317 (0.3%) of non-duplicate Enterobacteriaceae as carbapenemase producers, of which five harboured *bla*_NDM-1_ [*E. cloacae n *=* *2, *K. pneumoniae n *=* *2 (one with OXA-181), *E. coli n *=* *1] and four *bla*_OXA-48_ (*K. pneumoniae n *=* *3 and *S. marcescens n *=* *1).^43^ Surveillance of 100 isolates from 2011 mediated as part of the global SENTRY antimicrobial resistance surveillance programme identified three CPE (3%), two of which were NDM-1 producers (both *E. cloacae*; one from Bucharest and one from Cluj-Napoca), and one an OXA-48 producer (*K. pneumoniae*; from Bucharest).^44^ A third study focusing on surveillance in Cluj-Napoca, August 2011–November 2013,^45^ similarly identified ∼3% CPE prevalence (64/1903 isolates tested; all in individuals >16 years of age), with the earliest NDM-1 isolate an *E. cloacae* (26/9/2011) obtained from a patient in ambulatory care, and the first NDM-1 *S. marcescens* isolate reportedly from February 2013. Although the sampling in each of these studies was geographically and temporally restricted, the data from these studies and our survey would suggest that *bla*_NDM-1_ was present in non-*Serratia* spp. first, and subsequently emerged in *Serratia* spp.

Much of the recent surveillance of carbapenemase producers unsurprisingly focuses on those species of Enterobacteriaceae that cause most clinical disease, namely *E. coli* and/or *K. pneumoniae*. The most recent report of the European Antimicrobial Resistance Surveillance Network (EARS-Net) shows a significant increasing trend in carbapenem resistance in both *E. coli* and *K. pneumoniae* invasive isolates from Romania which is concerning.^46^ In addition, a recent European-wide survey (EuSCAPE) investigating clinical isolates of these two species suggested that in Romania most carbapenem resistance was attributable to clonal dissemination of a *K. pneumoniae* strain harbouring *bla*_OXA-48_.^47^ Our data and other recently published studies^19^^,^^45^^,^^48^^,^^49^ suggest that the epidemiology of carbapenemase producers is more complicated, and different mechanisms may be circulating in different species, with a degree of horizontal exchange of successful plasmids. In Czobor *et al.*,^48^ several NDM-1 isolates were harbouring IncFII_Y_ plasmids, consistent with these being pKOX_NDM1-like, although with the limited plasmid typing performed this cannot be confirmed. Surveillance is therefore difficult, needs to encompass asymptomatic individuals and a wide range of species types over time in order to be accurate, and needs to be high resolution and comparable across studies.

A limitation of our study is that our strain collection was not complete (i.e. did not include all NDM-positive isolates as well as consecutive non-NDM isolates representing background diversity for the two sites), but was predominantly focused on investigating NDM-1 strains collected as part of outbreak investigation, and sequencing of a small number of isolates that had been stored *ad hoc*. Although WGS is now relatively commonly used for molecular epidemiology and outbreak investigation, this is the first study to our knowledge to use WGS data to investigate the evidence for NDM-1 CPE transmission in Romania, and the mobile genetic elements involved in the acquisition and dissemination of *bla*_NDM-1_ in nosocomial *S. marcescens*. The use of long-read WGS data to fully resolve nine of the NDM-1 plasmid structures involved in this outbreak overcomes some of the pitfalls in investigating plasmid epidemiology using short-read WGS data, with which many plasmids are only reconstructed as partial fragments or contigs. However, evolutionary models to account for the genetic variation and plasticity observed in these plasmid structures (mutations, recombination, indels) have not yet been developed, and it was not possible to determine a clear sequence of plasmid transmission events by visual inspection alone, particularly in view of the fact that we are likely to have sampled only a proportion of potentially circulating plasmids. We also cannot exclude the possibility of multiple, discrete, pKOX_NDM1-like plasmid importation events into these hospitals.

In this context, our phylogenetic analysis of bacterial strains and high-resolution analysis of nine NDM-1 plasmids in circulating *K. pneumoniae*, *E. cloacae* and *S. marcescens* would be consistent with the transfer of pKOX_NDM1 from major non-*Serratia* spp. into several *S. marcescens* host strains. We found no evidence of any non-pKOX_NDM1-like plasmid backgrounds in the 34 NDM-1-positive Enterobacteriaceae sequenced across the study timeframe and two hospitals, which would be most consistent with the importation of this plasmid type and its local dissemination, as part of a pKOX_NDM1-like plasmid-associated outbreak, as opposed to the emergence of a separate successful Romanian NDM-1 plasmid. Further studies exploring the global diversity of pKOX_NDM1-like plasmids, which have been reported in several Enterobacteriaceae isolates from Canada,^50^ China,^51^ Australia/New Zealand^52^ and Saudi Arabia^53^ would be useful to identify the extent of transmission of this global, multi-species NDM-1 vector. 

## Supplementary Material

Supplementary Data

Supplementary Table S1
