# Supplementary material for: Illumina short-read and MinION long-read WGS to characterize the molecular epidemiology of an NDM-1 Serratia marcescens outbreak in Romania
Source: J Antimicrob Chemother. 2017 Dec 8;73(3):672–9. doi: 10.1093/jac/dkx456 (PMC5890751; doi:10.1093/jac/dkx456)
Supplement: Supplementary Data [file supplementary_data_dkx456.docx]

**Supplementary data**

Figure S1. Summary of genomic features of sequenced study isolates. Isolates boxed in blue are from Cluj-Napoca hospital, others from Targu-Mures hospital. Isolates 9275 and 17ES also contained the *bla*_OXA-48_ carbapenemase gene.

**Figure S2.** Long-read MinION results: read length distribution and other statistics

**Table S2.** Summary of MinION sequencing assemblies (n=10 isolates)

|  |  |  |  |  |  |  |  | NDM-1 contig information | | | |
| --- | --- | --- | --- | --- | --- | --- | --- | --- | --- | --- | --- |
| Sample | Species/ST | Coverage | Assembly size (bp) | # contigs | Mean contig size (bp) | Max contig size (bp) | Min contig size (bp) | NDM-1 contig | Length (bp) | Circularised | Overlapping size |
| 7209 | *S. marcescens* | 15 | 5638452 | 14 | 402747 | 1662736 | 11772 | tig00000046 | 93134 | yes | 5541 |
| 9580 | *S. marcescens* | 19 | 5643686 | 13 | 434130 | 1179329 | 57857 | tig00000056 | 115471 | yes | 9857 |
| 12TM | *S. marcescens* | 98 | 5509644 | 3 | 1836548 | 5285959 | 94286 | tig00000069 | 129399 | yes | 10353 |
| 14ES | *S. marcescens* | 40 | 5331861 | 6 | 888644 | 3599641 | 6400 | NA  (*bla*_NDM_-negative) | NA | NA | NA |
| 18ES | *K. pneumoniae*  ST307 | 12 | 5714026 | 10 | 571403 | 1478046 | 112277 | tig00000042 | 118336 | yes | 7904 |
| 5TM | *K. pneumoniae*  New ST | 47 | 6016823 | 11 | 546984 | 3036277 | 7956 | tig00000073 | 130514 | yes | 10108 |
| 20ES | *E. cloacae*  ST254 | 23 | 5617583 | 19 | 295662 | 3105465 | 5075 | tig00000068 | 120312 | yes | 9546 |
| 22ES | *E. cloacae*  ST90 | 98 | 5346364 | 13 | 411259 | 2478567 | 4970 | tig00000109 | 111157 | yes | 10764 |
| 4TM | *S. marcescens* | 48 | 5522902 | 5 | 1104580 | 4325511 | 16158 | tig00000109 | 130921 | yes | 11874 |
| 6TM | *K. pneumoniae*  New ST | 23 | 6015958 | 13 | 462766 | 2460992 | 6608 | tig00000045 | 128459 | yes | 8381 |
